# Supplementary material for: The interactome of intact mitochondria by cross-linking mass spectrometry provides evidence for coexisting respiratory supercomplexes
Source: Mol Cell Proteomics. 2017 Dec 8;17(2):216–32. doi: 10.1074/mcp.RA117.000470 (PMC5795388; doi:10.1074/mcp.RA117.000470)
Supplement: Supplemental Data [file supp_RA117.000470_133922_0_supp_23978_fzffwf.zip › spectra_annotation/mito_spectra_annotation/76-1-18-1-39-1.pdf]

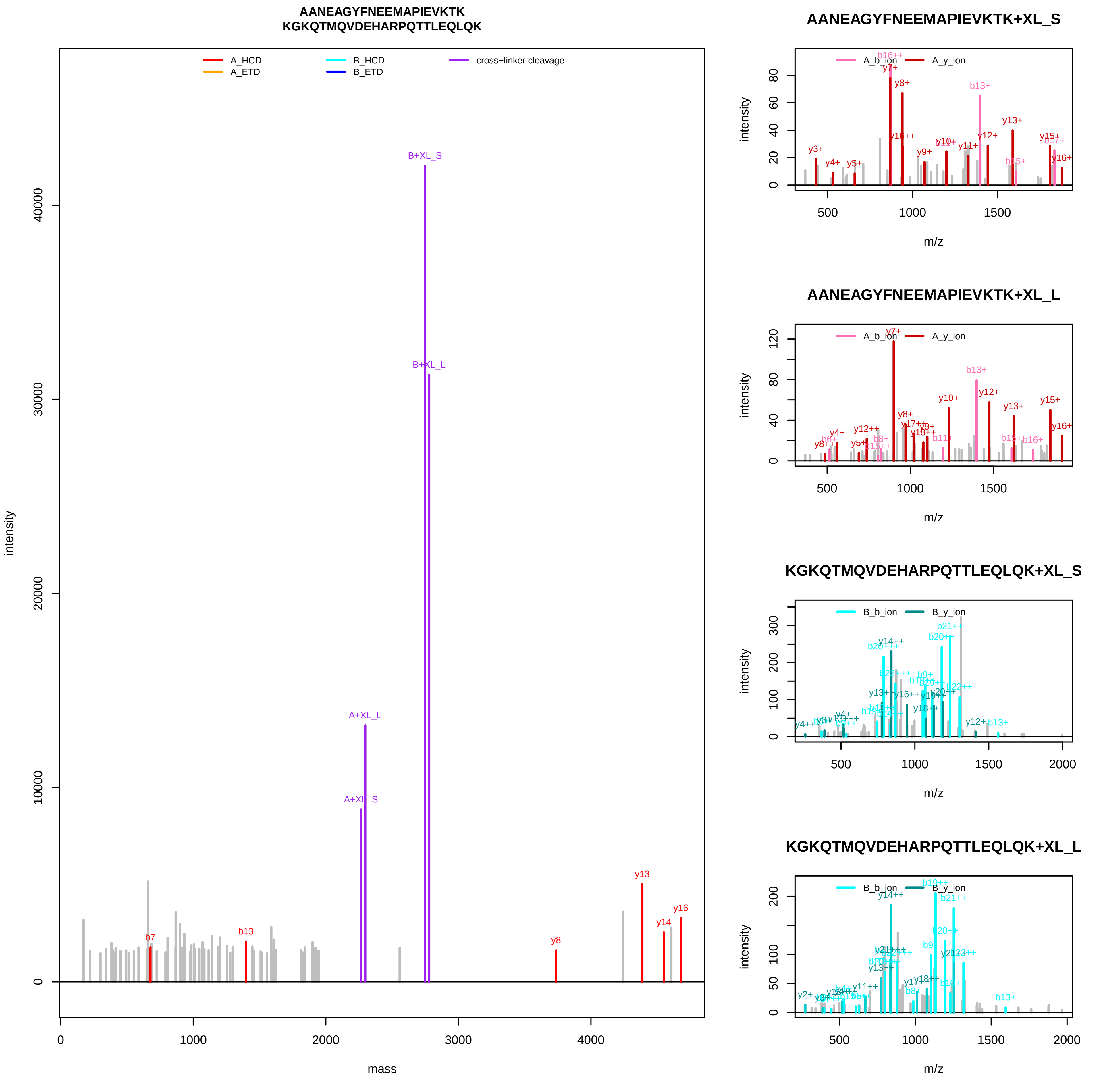

AANEAGYFNEEMAPIEVKTK
KGKQTMQVDEHARPQTTLEQLQK
A_HCD
A_ETD
B_HCD
B_ETD
cross−linker cleavage
B+XL_S
B+XL_L
A+XL_L
A+XL_S
b7
b13
y8
y13
y14
y16
intensity
mass
0
10000
20000
30000
40000
0
1000
2000
3000
4000
AANEAGYFNEEMAPIEVKTK+XL_S
A_b_ion
A_y_ion
y7+
y8+
b13+
y13+
y12+
y3+
y4+
y5+
y9+
y10+
y11+
y15+
y16+
AANEAGYFNEEMAPIEVKTK+XL_L
KGKQTMQVDEHARPQTTLEQLQK+XL_S
B_b_ion
B_y_ion
KGKQTMQVDEHARPQTTLEQLQK+XL_L
m/z
500
1000
1500
2000
